# In vivo visualization of age-related differences in the locus coeruleus

**DOI:** 10.1016/j.neurobiolaging.2018.10.014

**Published:** 2019-02

**Authors:** Kathy Y. Liu, Julio Acosta-Cabronero, Arturo Cardenas-Blanco, Clare Loane, Alex J. Berry, Matthew J. Betts, Rogier A. Kievit, Richard N. Henson, Emrah Düzel, Cam-CAN, Robert Howard, Dorothea Hämmerer

**Affiliations:** a Division of Psychiatry, University College London, London, UK; b Wellcome Centre for Human Neuroimaging, UCL Institute of Neurology, University College London, London, UK; c German Center for Neurodegenerative Diseases (DZNE), Magdeburg, Germany; d Institute of Cognitive Neurology and Dementia Research, Otto-von-Guericke-University Magdeburg, Magdeburg, Germany; e Institute of Cognitive Neuroscience, University College London, London, UK; f Camden & Islington NHS Foundation Trust, London, UK; g Medical Research Council Cognition and Brain Sciences Unit, University of Cambridge, Cambridge, UK; h Cambridge Centre for Ageing and Neuroscience (Cam-CAN), University of Cambridge and MRC Cognition and Brain Sciences Unit, Cambridge, UK

**Keywords:** Locus coeruleus, Noradrenergic system, Magnetic resonance imaging, Aging, Neuromelanin

## Abstract

The locus coeruleus (LC), the major origin of noradrenergic modulation of the central nervous system, may play an important role in neuropsychiatric disorders including Parkinson's disease and Alzheimer's disease. The pattern of age-related change of the LC across the life span is unclear. We obtained normalized, mean LC signal intensity values, that is, contrast ratios (CRs), from magnetization transfer–weighted images to investigate the relationship between LC CR and age in cognitively normal healthy adults (N = 605, age range 18–88 years). Study participants were part of the Cambridge Centre for Ageing and Neuroscience—an open-access, population-based data set. We found a quadratic relationship between LC CR and age, the peak occurring around 60 years, with no differences between males and females. Subregional analyses revealed that age-related decline in LC CR was confined to the rostral portion of the LC. Older adults showed greater variance in overall LC CR than younger adults, and the functional and clinical implications of these observed age-related differences require further investigation. Visualization of the LC in this study may inform how future scanning parameters can be optimized, and provides insight into how LC integrity changes across the life span.

## Highlights

• A quadratic relationship between locus coeruleus signal intensity and age was found.
• No differences between males and females were observed.
• Older adults had higher variance than younger adults.
• The functional and clinical implications of these changes require further research.

## 1 Introduction

The locus coeruleus (LC) is a cylindrical, hyperpigmented nucleus in the rostral pontine brainstem, and the major origin of noradrenergic neurons in the central nervous system. It plays an integral role in the regulation of arousal and autonomic function through connections with widespread areas of the brain and spinal cord (Samuels and Szabadi, 2008a), modulating wakefulness, pupil control, blood pressure, and temperature. It also plays a role in anxiogenesis and responses to fear and pain via connections with the amygdala (Samuels and Szabadi, 2008b), and has been implicated in attention, decision-making, and memory through connections with frontal and parietal cortices and the hippocampus (Sara, 2009).

**Fig. 1:**
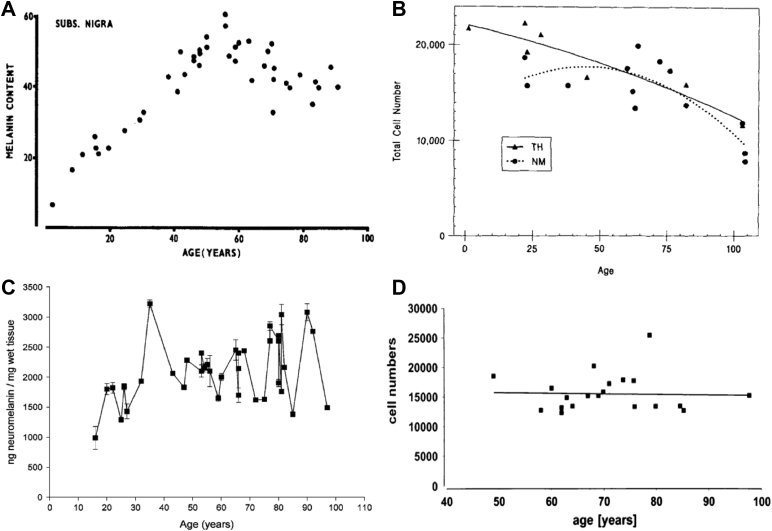
Postmortem studies have reported an inverted U-shaped (A, B) or linear (C) pattern of neuromelanin accrual in LC neurons, and a linear decline (B) or no change (D) in LC neuron number with increasing age. Graph A (reproduced with permission from Mann and Yates, 1974) shows mean melanin content of LC neurons against age from 45 human cases (age range 0–91 years). Participants did not have neurological illness or abnormal neuropathological findings. Graph B (reproduced with permission from Manaye et al., 1995) shows the total number of LC neurons (neuromelanin [NM] and tyrosine hydroxylase [TH] cell counts) on one side of the brain against age from 17 human cases (age range 1–104 years). Fourteen brains were from individuals with no known neurological or psychiatric disease, two had psychiatric illness and one had Down's syndrome. Graph C (reproduced with permission from [Zucca et al., 2006]) shows the mean neuromelanin content in LC neurons against age from healthy adults (age range 14–97 years) with no known neuropsychiatric or neurodegenerative disease. Graph D (reproduced with permission from Ohm et al., 1997) shows the stereological LC cell count from one side of the brain against age from 20 healthy adults (age range 49–98 years). Abbreviation: LC, locus coeruleus.

There is increasing interest in the development of in vivo techniques to assess the integrity of the LC after demonstration of early pathological change within this structure in neurodegenerative conditions such as Parkinson's disease (PD) (Braak et al., 2003) and Alzheimer's disease (AD) (Braak et al., 2011). In AD, levels of LC cytopathology (Grudzien et al., 2007) and cell loss (Kelly et al., 2017) were associated with cognitive impairment, and in vivo measures of LC integrity may have the potential to serve as a biomarker for presymptomatic stages of AD (Theofilas et al., 2017). Postmortem studies of healthy adults have reported a linear age-related decline in LC neuron numbers (Manaye et al., 1995) (see Fig. 1B), with some studies reporting cell loss mostly localized to the rostral part of the LC (Chan-Palay and Asan, 1989a, Manaye et al., 1995). However, several recent postmortem studies have found no age-related reduction in LC cell number in healthy adults (Mouton et al., 1994, Ohm et al., 1997, Theofilas et al., 2017) (see Fig. 1D). One explanation for these inconsistent findings could be that observed LC neuronal death in apparently cognitively normal older adults might represent the presymptomatic stage of a dementia, given that LC cell loss has been shown to occur early in the course of AD, PD, and Down's syndrome (German et al., 1992, Theofilas et al., 2017, Zarow et al., 2003), with disease-specific topographical patterns of cell loss (German et al., 1992). Thus, the development of a valid, in vivo technique to assess LC integrity is needed to investigate the potential role of this structure in healthy aging, preclinical and neurodegenerative disease patient groups.

The LC can be visualized in vivo using T1-weighted and magnetization transfer (MT)–weighted magnetic resonance imaging (MRI) (Nakane et al., 2008, Sasaki et al., 2006). These approaches, also known as “neuromelanin-sensitive” imaging, have attracted considerable interest as a potential means of quantifying neuronal loss in the LC in vivo. LC signal intensity has been shown to be associated with the location, density (Keren et al., 2009), and neuromelanin content of LC neurons in older adults (Keren et al., 2015). Neuromelanin is a by-product of noradrenaline production, and postmortem studies have found that neuromelanin accumulates inside the noradrenergic neurons of the LC with increasing age; however, the precise pattern of LC neuromelanin accrual over the life span remains unclear (see Fig. 1A–C). Several studies have reported an inverted U-shaped pattern of neuromelanin accrual across the life span, so that levels peak at around 60 years followed by a decline (Mann and Yates, 1974, Manaye et al., 1995), which may be related to age-related loss of neuromelanin-containing LC neurons. However, a more recent study reported a linear accumulation of LC neuromelanin with age, with no evidence of age-related decline (Zucca et al., 2006). Furthermore, it is also unclear how neuromelanin might interact with the cell in different contexts; neuromelanin scavenges metals such as iron and copper (Sasaki et al., 2008), which confers neuroprotective qualities, but it can also be toxic to aging neurons and contributes to neurodegeneration (Zecca et al., 2008). As the sample sizes of earlier postmortem studies were relatively limited and the sensitivity of the techniques used to detect neuromelanin may have differed, the precise relationship between neuromelanin content and neuronal health in the LC is still unclear. However, in vivo imaging studies have consistently found lower LC signal intensity in neurodegenerative disease patient groups, including AD and PD, compared with healthy controls (Liu et al., 2017), warranting further investigation of LC signal intensity as a potential biomarker of LC-noradrenergic system integrity.

To the best of our knowledge, only four in vivo imaging studies have reported age-related changes in LC signal intensity (Betts et al., 2017, Clewett et al., 2016, Dahl et al., 2018, Shibata et al., 2006), and one recent in vivo study found no age-related differences (Hämmerer et al., 2018). Two studies (Shibata et al., 2006, Clewett et al., 2016) reported age-related differences in mean LC signal intensity consistent with the inverted U-shaped pattern of neuromelanin accrual across the life span (Mann and Yates, 1974, Manaye et al., 1995). A third study reported a regional age-related increase in maximum (but not median) LC signal intensity ratio that was confined to the rostral third of the LC (Betts et al., 2017), which may indicate that age-related differences are confined to regions with a high density of LC pigmented cells, and the authors also speculated to a potential regional decrease in LC cell size thereby increasing cell volumetric mass density with age (German et al., 1988). By contrast, a fourth study (Dahl et al., 2018) found that relative to younger adults, older adults had higher peak signal intensity ratios in the caudal LC, with a trend for lower ratios in the rostral LC. As well as age-related changes, there may also be differences in LC signal intensity between males and females (Clewett et al., 2016), but this finding has not been consistently reported (Shibata et al., 2006). The relatively small sample sizes and different acquisition parameters and analyses used in these studies may have contributed to the inconsistent findings. Although a range of T1- and MT-weighted scanning protocols have been used to visualize the LC (Liu et al., 2017), an optimal scanning protocol to visualize or quantitatively measure the LC has not been established.

The Cambridge Centre for Ageing and Neuroscience (Cam-CAN) is a large-scale collaborative research project that aims to identify the neural mechanisms underlying successful aging in a population-based sample spanning the adult life span (Shafto et al., 2014). This database includes MT-weighted images of the brain from over 600 healthy participants aged between 18 and 88 years (Taylor et al., 2017) (see Methods section for a detailed scanning protocol).

We obtained normalized, mean LC signal intensities from previously acquired MT-weighted images from the Cam-CAN database (obtained from the Cam-CAN repository available at http://www.mrc-cbu.cam.ac.uk/datasets/camcan/) to investigate the relationship between LC signal intensity and age in cognitively normal healthy adults. By using the Cam-CAN database, we were able to use a much larger sample size than previous studies to investigate the relationship between LC signal intensity and age. We expected the LC signal intensity to increase with age in younger adults below the age of around 60 years, reflecting neuromelanin accumulation without substantial cell loss. However, the relationship between LC signal intensity and age in older adults from the age of around 60 years is less clear, because previous studies have reported conflicting findings and only involved relatively small samples. Visualization of the LC in this study was also compared with previous MT-weighted MRI studies of the LC, and its potential contribution toward an optimal scanning protocol for future studies is discussed.

## 2 Materials and methods

### 2.1 Participants

A population-based cohort of healthy adults (n = 708) was recruited as part of the Cam-CAN project (Shafto et al., 2014). Participants were cognitively healthy and excluded based on several criteria: Mini-Mental State Examination score <25 (consistent with the standard cutoff used for cognitive impairment screening); failing to hear a 35 dB 1 kH tone in either ear; poor English language skills (non-native or nonbilingual speakers); self-reported substance abuse, diagnoses of dementia/AD, PD, stroke, epilepsy, serious head injury, or other serious health conditions (e.g., major psychiatric conditions, a history of stroke or heart conditions); or MRI or magnetoencephalography contraindications (e.g., ferromagnetic metallic implants, pacemakers, or recent surgery). Usable MT-weighted imaging data were collected from 623 individuals (316 female, age range 18–88 years, mean = 54 years, SD = 18.6). Ethical approval for the study was obtained from the Cambridgeshire 2 (now East of England—Cambridge Central) Research Ethics Committee (reference: 10/H0308/50), and all participants provided written informed consent before the study.

### 2.2 Structural imaging protocol

Participants were scanned with a 3T Siemens TIM Trio System, using a 32-channel head coil. MT-weighted images for each participant were obtained from a 3D, MT-prepared spoiled gradient echo sequence with either repetition time (TR) = 30 ms or TR =50 ms (the TR =50 milliseconds sequences were used when the participant's SAR estimation for the TR = 30 milliseconds sequences exceeded the stimulation limits); echo time = 5 milliseconds; flip angle = 12°; field of view = 192 × 192 mm; voxel size = 1.5 mm isotropic; bandwidth = 190 Hz/px; acquisition time of 2 minutes and 36 seconds per sequence for TR = 30 milliseconds, and 4 minutes and 19 seconds per sequence for TR = 50 milliseconds. For MT weighting, a Gaussian shaped RF pulse with an offset frequency of 1950 Hz (bandwidth = 375 Hz, 500° flip angle, duration = 9984 microseconds) was used. A 3D T1-weighted structural image was also acquired for each participant using a magnetization-prepared rapid gradient echo sequence with the following parameters: TR = 2250 milliseconds, echo time = 2.00 milliseconds, inversion time = 900 milliseconds; flip angle = 9°; field of view = 256 × 240 × 192 mm; voxel size = 1 mm isotropic; generalized autocalibrating partial parallel acquisition acceleration factor = 2; acquisition time of 4 minutes 32 seconds. MT- and T1-weighted images were then processed and analyzed as described in the following.

### 2.3 Spatial normalization and coregistration

**Fig. 2:**
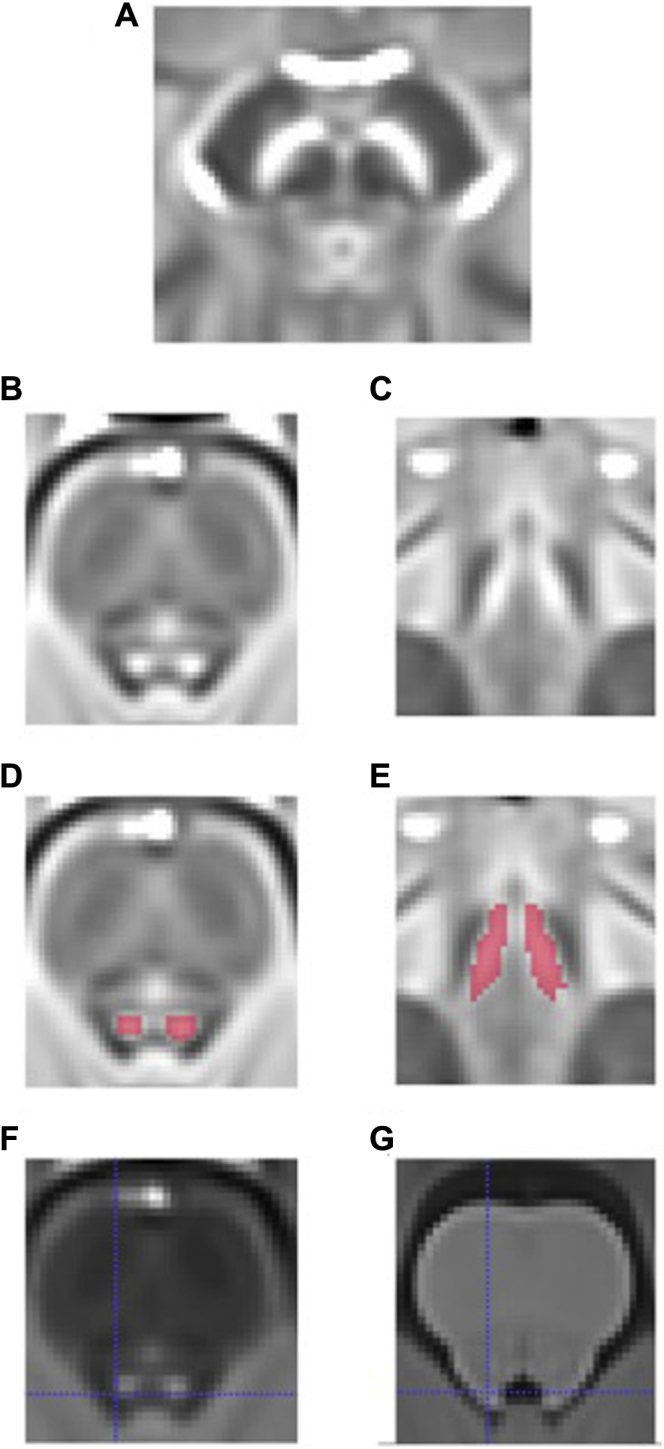
Cam-CAN MT-weighted group template images showing LC (axial B,D, coronal C,E) with LC segmentations (D,E in red). A multivariate template approach was used to create an MT-weighted (F) and T1-weighted (G) group image. The substantia nigra could be visualized on the MT-weighted group template (A). In (B) and (F), two areas of signal hyperintensity are visible which correspond to the neuroanatomical positions of right and left LC in the lateral floor on the 4th ventricle in (G). The left LC can be seen at the crosshair in (F) and its position in (G) is indicated by the corresponding crosshair. Segmentation of these areas was performed on the axial slices on the MT-weighted template (D). The substantia nigra, a region with similar macromolecular properties to the LC, could also be visualized as two areas of signal hyperintensity on the MT-weighted group template (A). Abbreviations: LC, locus coeruleus; Cam-CAN, Cambridge Centre for Ageing and Neuroscience; MT, magnetization transfer. (For interpretation of the references to color in this figure legend, the reader is referred to the Web version of this article.)

Before spatial normalization, single-subject MT-weighted images were upsampled to 0.8-mm isotropic resolution to improve visualization of the LC. MT-weighted images were then bias-corrected (N4-ITK) (Tustison et al., 2010) and spatially normalized to a studywise space using a highly iterative coregistration routine available from the ANTS v2.1 software package (http://stnava.github.io/ANTs). On neuromelanin-optimized, MT-weighted MRI scans, the LC appears as hyperintense voxels bilaterally on the lateral floor of the fourth ventricle. To confirm the anatomical location of the LC, a multicontrast template was created from the upsampled MT-weighted and T1-weighted images using the default settings in the “antsMultiVariateTemplateConstruction2.sh” script in ANTS v2.1. This approach uses the two modalities to drive registration to create a group average image for each input modality, i.e., a T1- and MT-weighted group average image (see Fig. 2F and G). Both images were coregistered using “antsRegistrationSyNQuick.sh” script in ANTS v2.1, and the T1-weighted image was used to confirm the location of the LC mask with greater precision compared with the lower resolution MT-weighted images.

### 2.4 LC segmentation and signal intensity extraction

**Fig. 3:**
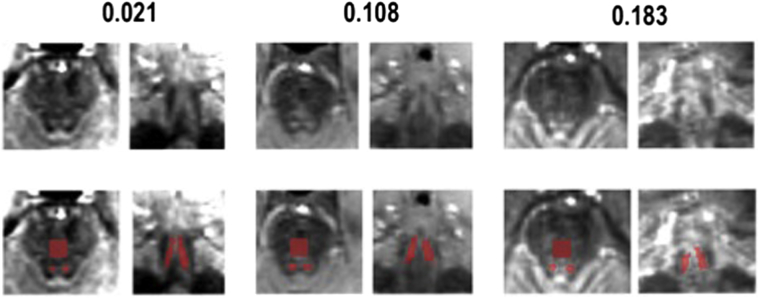
Cam-CAN MT-weighted images from three individual participants with LC and reference region (pons) segmentations (second row, red). The number above each image is the mean LC CR value for an individual participant, demonstrating that the LC can be visualized on individual scans as two areas of signal hyperintensity (first row) across the range of LC CR values, shown in axial (left) and coronal (right) views. In the second row, the large red square represents the reference region (pons). These images for each participant were visually assessed to ensure correct identification of the LC and to detect any significant movement artifacts. Abbreviations: CR, contrast ratio; LC, locus coeruleus; Cam-CAN, Cambridge Centre for Ageing and Neuroscience; MT, magnetization transfer. (For interpretation of the references to color in this figure legend, the reader is referred to the Web version of this article.)

The left and right LC were each manually segmented on the studywise MT-weighted template independently by two authors (KL and DH) using the software package ITK-Snap (Yushkevich et al., 2006). The LC mask was defined as the conjunction of labeled voxels from the two raters (see Fig. 2D and E) and inter-rater agreement was assessed. The LC mask was warped back to the individual native space using ANTS v2.1 (Avants et al., 2011), and visually assessed for each participant to ensure correct identification of the LC in individual scans (see Fig. 3). Individual MT- and T1-weighted scans were also visually inspected for movement artifacts independently by two authors (KL and DH) and 17 data sets were excluded as the motion artifacts were judged to be significant by both authors. After the mean signal intensity in the LC masks from the remaining 605 scans was extracted using an in-house script written in MATLAB (2014b; Mathworks Inc, Natick, MA, USA) software, an additional participant was subsequently excluded after initial analysis revealed a negative mean LC contrast ratio (CR) value, which on further inspection was because of previously undetected motion in the brainstem region (for the excluded 18 participants, age range was 20–80 years, mean [SD] = 59 [22] years). Thus, 605 participants (97% of original sample) were included in the final analysis.

To account for intersubject variability in overall signal intensity, mean signal intensities in the left and right LC were assessed relative to a reference region in the pons (10 × 10 voxels or 8 × 8 mm wide) on the studywise template, placed automatically in the middle slice of the LC mask in the z direction, halfway between the left and right LC in the x (left-right) direction, and 5 voxels (2 mm) above the LC mask in the y (rostrocaudal) direction (see Fig. 3). The size of the reference region was previously chosen in two published studies (Clewett et al., 2016, Hämmerer et al., 2018) to avoid capturing low signal intensities in the ventral tip of the fourth ventricle and the darker medial lemniscus/pons. Normalization of the LC signal to a reference region (pons) to calculate the CR has been the most commonly used method to report findings in previous studies (Liu et al., 2017), and mean signal intensity values were extracted to calculate normalized LC signal intensities (CR) using the following formula:

$$CR=\frac{\left(S_{LC}-\;S_{ref}\right)}{S_{ref}}$$

where S_LC_ represents the signal intensity of the LC and S_ref_, the signal intensity of a reference region. This was also the most commonly used CR formula from previous studies (Liu et al., 2017).

### 2.5 Statistical analysis

Both linear and quadratic regression analyses with alpha level of 0.05 were performed and compared using the Akaike information criterion (AIC) to determine the best model to fit the pattern of age-dependent differences in LC CR. This was followed by a post hoc “two-lines” test (Simonsohn, 2017) to test the validity of a U-shaped relationship. Difference in mean LC CR between males and females was analyzed with an independent Student's t-test and a two-sided Bayesian independent *t*-test with a default Cauchy prior width of $r=\sqrt{\left[2\right]/2}$ (Morey et al., 2011) (the Bayes factor provides several advantages over the *p*-value, i.e., it compares the likelihood of both the null and alternative hypotheses and thus can be used to corroborate the null hypothesis [Dienes, 2014]). Age-related differences in reference region (pontine) signal intensity were also assessed, controlling for the effect of the two TR parameters (30 or 50 ms). Multiple regression of age on LC CR was subsequently performed, with age modeled by linear and quadratic terms, reference region (pontine) signal intensity and TR parameter as covariates, and an age-sex interaction term. High multicollinearity, or a high degree of correlation between two or more predictor variables, can decrease the reliability of the results in a regression model. Therefore, the degree of multicollinearity between variables was measured by calculating the variance inflation factor (VIF), with a VIF >5 indicating high correlation.

As secondary analyses, regional age-related differences in the LC were compared (using the same methods described previously) after dividing each participant's LC into a rostral and caudal region either side of the median voxel along the z-axis. Differences in rostral and caudal LC CR between older and younger adults were measured using Welch's t-tests. The mean number of voxels of the LC was related to age (linear and quadratic regressions) and TR group (30 ms or 50 ms), and the left and right LC were compared in terms of the mean number of voxels and maximum signal intensity ratios using paired t-tests. The maximum LC CR was calculated to allow cross-study comparison, as prior studies have also used this measure to investigate left-right (Betts et al., 2017, Tona et al., 2017) and rostrocaudal (Betts et al., 2017, Dahl et al., 2018) differences.

## 3 Results

### 3.1 LC segmentation and signal intensity extraction

**Fig. 4:**
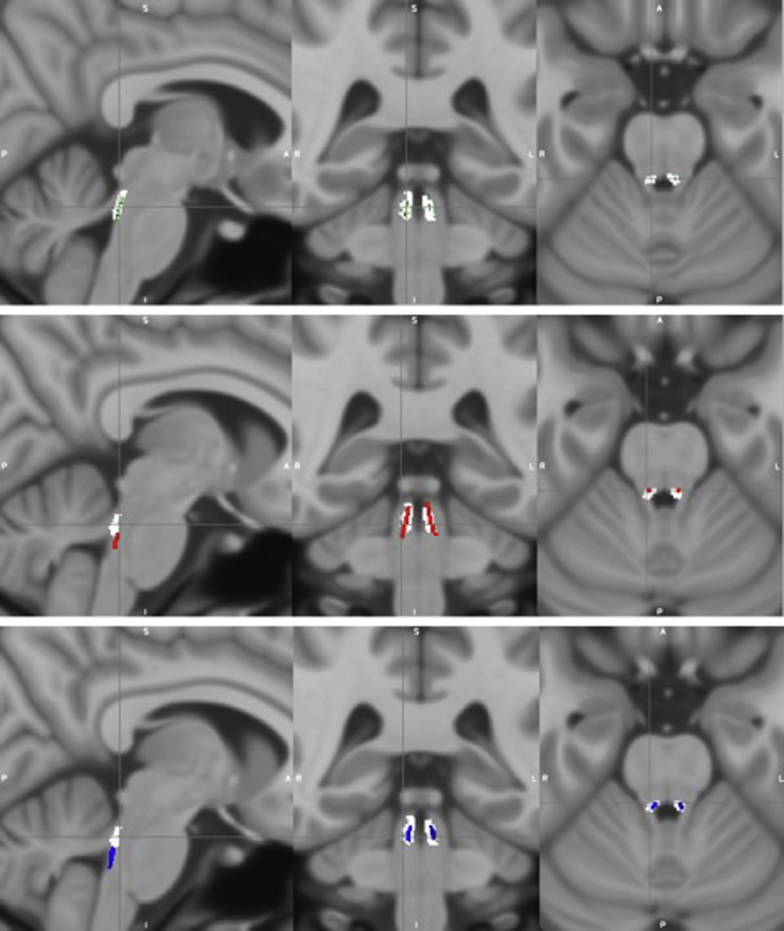
Spatial overlap between the Cam-CAN LC mask and three other published LC masks in MNI space. The Cam-CAN LC mask (white) showed spatial overlap with LC masks from three previous studies (Betts et al., 2017, Dahl et al., 2018, Keren et al., 2009), overlapping 94% relative to the Dahl mask (top row, green), 48% relative to the Keren 1SD mask (middle row, red), and 19% relative to the Betts mask (bottom row, blue). As was the case for the Dahl mask (Dahl et al., 2018), it can be observed that the Cam-CAN LC mask does not extend as far caudally as did the Keren and Betts masks, explaining the relatively lower overlap, and the Betts mask (which had the lowest overlap) did not extend as far rostrally as did the other masks. Abbreviations: LC, locus coeruleus; Cam-CAN, Cambridge Centre for Ageing and Neuroscience. (For interpretation of the references to color in this figure legend, the reader is referred to the Web version of this article.)

Two areas of signal hyperintensity were observed on the MT-weighted group template, which corresponded to the shape and neuroanatomical locations of the right and left LC when compared with the T1-weighted group image created using the multivariate template approach (Fig. 2G). Notably, the LC could also be visualized on individual scans across the range of LC CR values (Fig. 3). The inter-rater agreement, or percentage overlap, for the LC segmentations was 87% for rater DH and 80% for rater KL. The resulting LC mask had a maximum dorsoventral and mediolateral extension of 3.2 mm, a rostrocaudal extension of 12.8 mm, and consisted of 333 voxels in total, which equated to approximately 171 mm^3^ (approximately 85 mm^3^ for each LC). These measurements were close to previously published ex vivo dimensions (14.5 mm × 2.5 mm × 2 mm and an estimated volume of around 70 mm^3^ for each LC) (Fernandes et al., 2012). To allow comparison between the Cam-CAN LC mask and previously published LC masks (Betts et al., 2017, Dahl et al., 2018, Keren et al., 2009), the MT-weighted template was coregistered to the MNI152 space (using the “antsRegistrationSyNQuick.sh” routine in ANTS v2.1, with “rigid + deformable syn” as transformation type) and the obtained transformation matrices were applied to warp the mask to MNI space. The Cam-CAN LC mask percentage spatial overlap was 94%, 48%, and 19% relative to the Dahl, Keren (1SD), and Betts mask volumes, respectively. As was reported to be the case with the Dahl mask (Dahl et al., 2018), it was observed that the Cam-CAN LC mask did not extend as far caudally as did the Keren and Betts masks, explaining the relatively lower overlap with these masks (see Fig. 4). To illustrate this, after dividing the Keren mask into three equally sized (upper, middle, and lower) sections, the resulting Cam-CAN LC mask percentage spatial overlap values relative to these areas were 67%, 89%, and 7%, respectively. The overlap of the rostral Cam-CAN mask relative to the upper third of Keren's mask was 67%, and the overlap of the caudal Cam-CAN mask relative to the middle third of Keren's mask was 64%. The Betts mask (which had the lowest overlap) also did not extend as far rostrally as did the other masks. The substantia nigra, a region with similar macromolecular properties to the LC, could also be visualized as two areas of signal hyperintensity on the MT-weighted template and MT-weighted multivariate group image (Fig. 2A), providing further support for the validity of the observed LC signal.

### 3.2 Relationship between LC CR and age

**Fig. 5:**
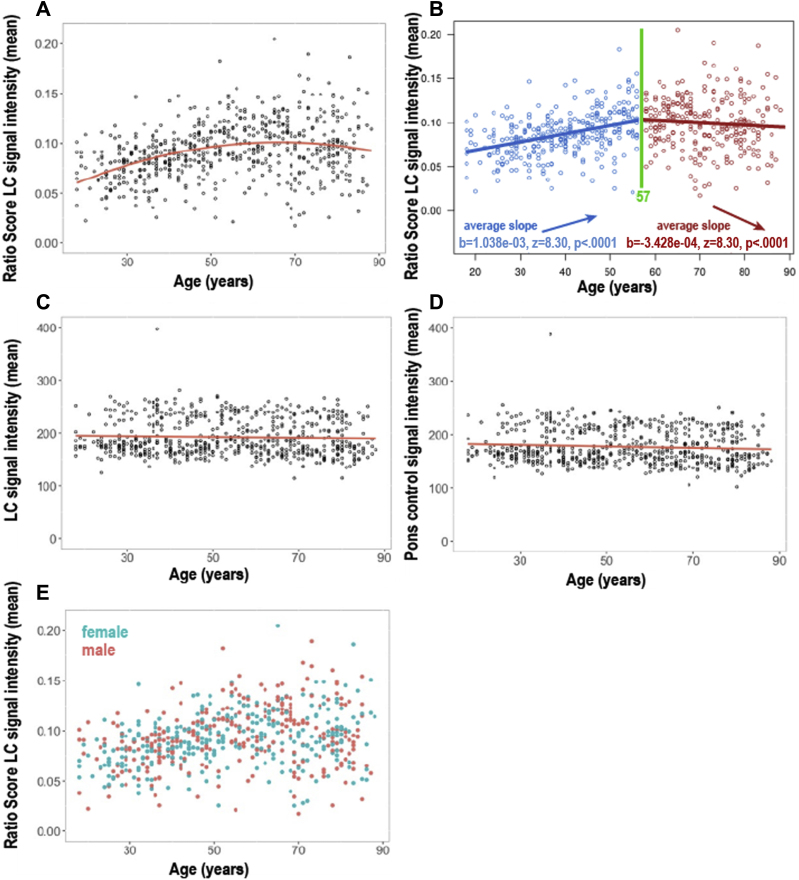
Graphs showing the relationship between mean LC CR (A, B, E), raw LC mean signal intensity (C), or mean pontine reference region signal intensity (D) and age in years. Unfilled circles (A, B, C, D) represent individual data points (n = 605); solid red circles represent males (n = 295) and solid blue circles represent females (n = 310) (E). In A, a quadratic model (adjusted R^2^ = 0.126, *p* < 0.001) provided a better fit than a linear model (adjusted R^2^ = 0.086, *p* < 0.001) according to the AIC. The quadratic relationship remained significant (adjusted R^2^ = 0.181, *p* < 0.001) after controlling for reference region signal intensity and TR scan parameter (30 or 50 ms). In (B), a post hoc “two-lines” test (proposed to be a valid alternative to testing a U-shaped relationship) provided a breakpoint of 57 years but the second slope was not individually significant (*p* = 0.92); not fully supporting the presence of a U-shaped relationship between LC CR and age. This may have been related to a smaller age range and higher variance (F = 1.46, *p* < 0.001) in the “older” (>57 years) versus “younger” group (<57 years). C shows a nonsignificant, weak, negative linear relationship between the raw LC signal intensity mean scores and age (r = −0.039, adj R^2^ = 0.0002, *p* = 0.341) and (D) shows the observed significant negative linear relationship between reference region (pontine) signal intensity and age (r = −0.1, adj R^2^ = 0.005, *p* = 0.049). In (E), no significant difference in mean LC CR was found between males (n = 295, solid red circles) and females (n = 310, solid blue circles) using a Bayesian approach. Abbreviations: LC, locus coeruleus; CR, contrast ratio; TR, repetition time; AIC, Akaike information criterion. (For interpretation of the references to color in this figure legend, the reader is referred to the Web version of this article.)

Fig. 5 (A) shows the mean LC CR for each participant plotted against age (n = 605). A quadratic model (adjusted R^2^ = 0.126, *p* < 0.001) was a better fit for the data compared with a linear model (adjusted R^2^ = 0.086, *p* < 0.001) according to the AIC (AIC_diff_ = 26). However, the validity of testing U-shaped relationships (i.e., an increase followed by a decrease) with a quadratic regression has recently been contested (Simonsohn, 2017), so a post hoc “two-lines” test (proposed as a valid alternative [Simonsohn, 2017]) was conducted (Fig. 5B). The breakpoint for “low” versus “high” age groups using this test was 57 years, and although the two slopes were of opposite sign, the downward slope was not individually significant (*p* = 0.92), thus not conclusively supporting an inverted U-shaped relationship between whole LC CR and age in this sample. The variance in the older group (>57 years) was significantly higher than that in the younger group (<57 years) (F = 1.46, *p* < 0.001).

No significant difference in mean LC CR between males (M = 0.094, SD = 0.029) and females (M = 0.092, SD = 0.026) was found; t(603) = 1.46, *p* = 0.144, Bayes factor = 3.9 in favor of the null hypothesis (effect size d [95% CI] = 0.12 [−0.04, 0.28]), and this null effect was also observed within subgroups of older (>57 years) and younger (<57 years) individuals. The TR parameter was reported for 599 of the total 605 participants (n = 369 for 30 ms and n = 230 for 50 ms) and the two TR groups differed significantly in mean age (t = −2.6[523], *p* < 0.01), mean LC CR (t = 3.8[462], *p* < 0.001) and mean reference region signal intensity (t = −30.8[327], *p* < 0.001). A negative linear relationship between the reference region (pons) signal intensity and age was observed (β = −0.08, adjusted R^2^ = 0.005, *p* = 0.049, see Fig. 5D) which remained significant after controlling for TR (β = −0.17, adjusted R^2^ = 0.693, *p* < 0.001). The quadratic relationship between age and mean LC CR also remained significant after adjusting for reference region (pontine) signal intensity and TR (30 or 50 ms) in a multiple regression analysis that included a second-degree polynomial expansion of age and an age-sex interaction term (adjusted R^2^ = 0.144, F(4,594) = 26.1, *p* < 0.001). No significant age-sex interaction effect was observed. Tests for multicollinearity indicated that only a low level of multicollinearity was present (VIF = 1.10 for age, 3.26 for reference region signal intensity, and 3.28 for TR).

**Fig. 6:**
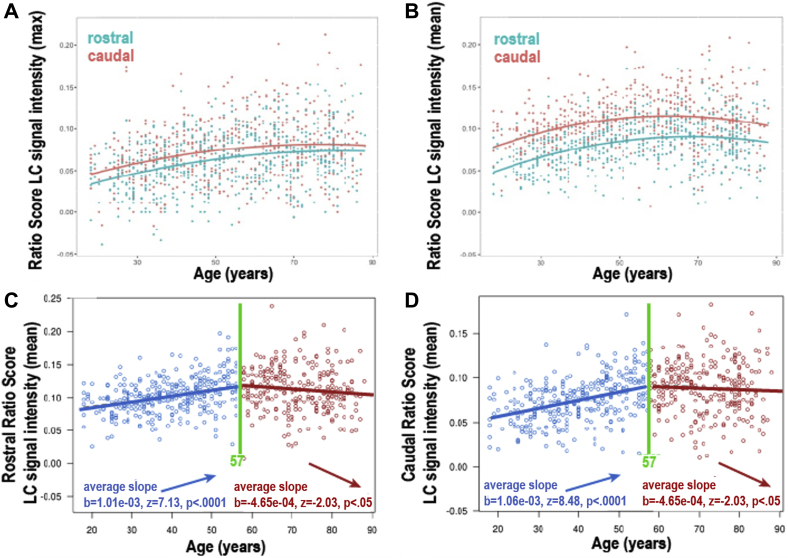
Graphs showing regional age-related differences in LC CR, plotting maximum (A) or mean (B, C, D) LC CR for rostral/caudal regions of LC and age in years. Solid red and blue circles (A and B) represent individual data points for rostral and caudal LC CR values, respectively. Unfilled circles (C and D) represent individual data points (n = 605). Age-related differences were observed for maximum (A) and mean (B) LC CR within rostral and caudal regions of LC, and the rostral region showed significantly higher LC CR values than the caudal region. Quadratic models were a better fit for the data compared with a linear model according to the AIC. A subsequent two-lines test revealed an inverted U-shaped relationship (with breakpoints shown in green) between mean LC CR and age in the rostral region but not in the caudal region, as both the upward (*p* < 0.001) and downward (*p* = 0.0426) slopes for the former reached statistical significance. Abbreviations: LC, locus coeruleus; CR, contrast ratio; AIC, Akaike information criterion. (For interpretation of the references to color in this figure legend, the reader is referred to the Web version of this article.)

After dividing each participant's LC into a rostral and caudal region, the maximum and mean LC CR from these regions were plotted against age (Fig. 6A and B). Because the Betts and Keren LC masks were observed to extend more caudally than the Cam-CAN LC mask (Fig. 4), the latter is likely to be capturing the rostral and middle portion of the LC. Therefore, the caudal region of the LC mask in this study is likely to represent the middle portion of the actual LC. On average, the maximum LC CR was significantly higher in the rostral (mean = 0.072, SD = 0.037) than the caudal region of the LC mask (mean = 0.062, SD = 0.03); t(604)= 10.4, *p* < 0.001) (Fig. 6A). Older adults (>57 years) had higher maximum, mean and median LC CR values in the rostral (maximum: t(542) = 5.28, *p* < 0.001; mean: t(520) = 3.92, *p* < 0.001; median: t(516) = 3.52, *p* < 0.001) and caudal (maximum: t(577) = 6.69, *p* < 0.001; mean: t(539) = 6.63, *p* < 0.001; median: t(529) = 6.66, *p* < 0.001) regions compared with younger adults (<57 years). As for the whole LC, quadratic models were a better fit for the rostral and caudal LC CR data than linear models according to the AIC. Subsequent two-lines tests revealed that only the rostral region showed an inverted U-shaped relationship between mean LC CR and age (Fig. 6C), with both the upward (*p* < 0.001) and downward (*p* = 0.0426) slopes reaching statistical significance. The mean number of voxels of LC for the 605 participants was 310 voxels (SD = 45.5). There was no significant relationship between the total number of voxels and age (linear [*p* = 0.08, Bayes factor = 2.4 in favor of null] or quadratic [*p* = 0.18] regressions) or between number of voxels and TR group (30 or 50 ms) (*p* = 0.77, Bayes factor = 10 in favor of the null hypothesis). On average, the left LC had a significantly higher number of voxels (mean = 162, SD = 26.0; t(622) = 17.5, *p* < 0.001) and higher maximum CR (mean = 0.071, SD 0.036; t(604) = 7.48, *p* < 0.001) compared with the right (mean voxels = 148, SD = 24.0; mean maximum LC CR = 0.063, SD = 0.037).

## 4 Discussion

With 605 healthy participants aged between 18 and 88 years, this is the largest in vivo study to investigate the relationship between age and LC signal intensity in a cross-sectional sample across the life span. It is also the first study to use existing MT-weighted scans from an open-access database (Cam-CAN) that had used scanning parameters not specifically selected to investigate and visualize the LC.

Previous studies have reported an inverted U-shaped pattern (i.e., an increase up to around 60 years followed by a decrease) between LC signal intensity and age in vivo (Shibata et al., 2006) and LC neuromelanin and age ex vivo (Mann and Yates, 1974). Consistent with these studies, we found that a quadratic model was a better fit for the data, with the peak occurring around 60 years. Our findings support the concept that LC signal intensity increases with age in younger adults below the age of around 60 years because of neuromelanin accumulation without substantial cell loss. However, as the precise relationship between age-related differences in LC CR and the morphology and number of LC neurons was not investigated, we cannot prove this nor exclude the possibility of age-related shrinkage of pigmented cells. We also observed that LC CR no longer increases from around 60 years, which could be because of an overall reduction in neuromelanin content related to loss of neuromelanin-containing noradrenergic neurons in the LC. Our analyses revealed that within the rostral LC, there was an inverted U-shaped relationship between mean LC CR and age, but our data precluded conclusive evidence for the presence of overall age-related decline in LC CR in the caudal region of the mask and the LC as a whole. This is because, unlike previous studies, we used a post hoc “two-lines” test to assess the validity of a U-shaped relationship, and found that the downward slope was only statistically significant for the rostral LC (*p* = 0.04), and not the more caudal region (*p* = 0.25) or whole LC (*p* = 0.92). Although this was not the primary hypothesis of this study, it is consistent with what we would expect from previous postmortem studies, which found greater age-related cell loss within the rostral compared with more caudal regions of the LC (Chan-Palay and Asan, 1989a, Manaye et al., 1995). Notably, this is also the pattern of LC cell loss that occurs in AD (Chan-Palay and Asan, 1989b, German et al., 1992). Animal studies have indicated that rostral LC cells project to the cerebral cortex and forebrain (including hippocampus) structures, whereas caudal LC neurons innervate spinal cord and cerebellum (Loughlin et al., 1986, Schwarz and Luo, 2015, Ungerstedt, 1971). In the forebrain, noradrenaline plays a role in modulating cognitive functions such as attention, learning, and memory; thus, our findings also support observations that age-related differences in the noradrenergic system affect these cognitive functions in older adults, for example, memory performance (Dahl et al., 2018, Hämmerer et al., 2018).

Our study found no significant difference in LC CR between males and females using a Bayesian approach in a sample of 605 participants. Previous studies have reported conflicting findings on this, which may have been related to their smaller sample sizes (<70 participants) and/or different scan parameters and analyses (Clewett et al., 2016, Shibata et al., 2006).

Conflicting findings have also been reported on the precise topographical pattern of age-related differences in LC signal intensity ratios, as one study found higher maximum (but not median) rostral LC CR in older adults (Betts et al., 2017) while another found higher maximum LC CR in the caudal LC in this age group (Dahl et al., 2018) compared with younger adults. Using a much larger sample, our study found that on average, older adults (>57 years) had significantly higher maximum, mean, and median LC CR values in the rostral and caudal portions of the LC mask compared with younger adults (<57 years).

We also observed that, on average, the left LC had a significantly higher number of voxels and higher maximum LC CR compared with the right. A lateralization effect has previously been reported, but so far the findings have been inconsistent; one recent study found that the right LC was larger and had higher maximum LC CR than the left (Tona et al., 2017), while another study found higher maximum and median LC CR in the left versus right LC (Betts et al., 2017). One possible explanation for the CR difference between right and left LC could be variations in radiofrequency asymmetry related to different scanners; however, when we analyzed a second pontine reference region consisting of right and left subregions (see in the following and in Supplementary Materials), we found the opposite lateralization effect in raw signal intensity (i.e., right > left), supporting the concept that the observed lateralization in LC CR represents functional lateralization rather than a technical effect. Previous functional MRI studies that have reported activation location coordinates for left and/or right LC support the possible presence of functional lateralization, reporting left LC activation (N = 15), right LC activation (N = 12), and bilateral activation (N = 8) associated with a wide range of conditions and cognitive tasks (Liu et al., 2017). There does not appear to be a clear distinction between conditions or tasks associated with left, right, or bilateral LC activation, although some cognitive functions were consistently associated with right or left LC. For example, stress and pain activated right LC in some studies and left LC in others, and three studies that used tasks to probe attention consistently reported right LC activation, and three studies that investigated decision-making and novelty consistently reported left LC activation. Further studies are needed to investigate the observed lateralization effect in the LC and pons.

As with previously reported postmortem studies, it is not known whether our findings represent the effects of healthy aging alone, or whether they are affected by the additional presence of individuals with presymptomatic pathological conditions such as AD. Older adults showed higher interindividual variance in whole LC mean signal intensity CRs compared with younger adults, which may have been related to differences in cognitive reserve (education, occupational attainment, and verbal intelligence) (Clewett et al., 2016) and memory performance (Hämmerer et al., 2018), both of which have been positively related to LC signal intensity in older adults. Although our study used a much larger sample size than previous studies, it is possible that the power required to show a significant age-related decline in whole LC signal intensity in the older group (>57 years) was limited by higher variance (F = 1.52, *p* < 0.001) and reduced age range compared with younger adults (<57 years), thus a larger sample and/or recruitment of more participants over 90 years may have provided more precision to conclusively define age-related differences in the LC. Participants' cognitive and behavioral outcomes on tasks known to be dependent on functional integrity of the noradrenergic system (e.g., emotional regulation/memory, inhibition, and attention) may help to clarify the functional significance of these observed age-related differences in older adults, and this is an area that we plan to investigate in a subsequent study. Follow-up of the participants would help to identify any who were at the early and presymptomatic stage of a neurodegenerative disorder (despite scoring above the conventional Mini-Mental State Examination cutoff for dementia at the time of scanning), which might have influenced the LC signal intensity rather than the effects of increasing age alone.

The observed age-related differences in LC CR (Fig. 5, Fig. 6) have potential implications for sample size calculations in future studies, as the statistical power needed to show age-related changes may vary depending on where recruited participants lie on particular sections of the quadratic curve, and which regions of the LC are measured. For example, our study supports the findings of a recent in vivo cohort study (Clewett et al., 2016), which reported higher mean LC signal intensity in older adults (mean age 67, range 58–75 years) compared with younger adults (mean age 24, range 18–34 years). Comparison of Cam-CAN participants within these age ranges (mean age in older adults, 66 years; in younger adults, 28 years) also showed that the older group had a significantly higher mean LC signal intensity than the younger group (t = 8.06, *p* < 0.001). Owing to the observed quadratic relationship between LC CR and age, division of a sample into older and younger participant groups to investigate age-related changes in the LC may obscure potential age-related decline in signal intensity in the over 60s.

Normalization of LC signal intensity to a reference region aims to reduce interindividual variability and to facilitate across-study comparison, as long as the same calculation is used. However, a potential limitation with this approach is that the reference region may also be differentially affected in healthy aging and disease states. We and others (Clewett et al., 2016, Keren et al., 2009) found an age-related decrease in signal intensity in the pontine tegmentum, but the underlying basis for this observation is unknown and it is not a consistent finding (Betts et al., 2017). The effect found in this study contributed to some but not all of the observed age-related increase in LC CR in younger adults, and would not have accounted for the overall nonlinear relationship and downward slope in LC CR from around the age of 60 years. To explore whether a different reference region would change our findings, we segmented a larger 3D pontine reference region (this pons mask consisted of 144 voxels) consisting of right and left subregions (Supplemental Fig. 1). There was no significant relationship between age and signal intensity in this reference region (r = −0.03, *p* = 0.45), and the right pontine reference region had a higher raw mean signal intensity than the left (t(604) = −17.4, *p* < 0.001). Similar findings were obtained; age-related differences in LC CR using this new reference region were better explained by a quadratic model (adjusted R^2^ = 0.02, *p* = 0.002) (Supplemental Fig. 2) than a linear model (adj R^2^ =0.0004, *p* = 0.26), and a subsequent two-lines test (Supplemental Fig. 2) showed that both the upward and downward slopes were significant (*p* = 0.02 and *p* = 0.006, respectively) (see Supplemental Materials for full data).

In this study, the LC could be clearly seen on the MT-weighted group template. The most common scanning technique used in structural MRI studies to investigate the LC has been the use of T1-weighted rapid acquisition with refocused echoes pulse sequences, also known as turbo/fast spin echo (Liu et al., 2017). Both T1-shortening and MT effects have been considered as the source of neuromelanin contrast (Trujillo et al., 2016). To the best of our knowledge, only three previous imaging studies have used an explicit MT presaturation pulse to visualize the LC in vivo, in combination with 2D or 3D gradient echo sequences (Chen et al., 2014, Langley et al., 2016, Priovoulos et al., 2017). Compared with the published images from these studies, the LC signal from the MT-weighted Cam-CAN group template appears to have higher signal contrast and lower spatial resolution, leading to better visualization but potentially lower precision for manual segmentation. However, a direct comparison with these studies is precluded due to the fact that our group template was constructed using over 600 scans, whereas previous studies used much smaller sample sizes. The Cam-CAN LC mask showed good overlap relative to the previously published Dahl mask volume (94%) and the rostral/middle portions of the Keren and Betts masks. The greater caudal extension of the Keren/Betts LC masks compared with our mask could be because the relatively lower resolution of the Cam-CAN scanning sequence could not sufficiently identify this portion, which has previously been reported to show a less reliably observable signal (Betts et al., 2017). The Cam-CAN LC mask, warped to 0.5 mm^3^ MNI space, is available to download from the online Supplementary materials. The acquisition times of the Cam-CAN MT-weighted sequences were under 5 minutes, which would be more acceptable for translation to clinical populations such as AD and PD compared with the other studies that acquired MT-weighted images in over 12 minutes. A consensus scanning protocol to investigate the LC has not yet been achieved, thus the Cam-CAN scanning parameters reported in this study may inform how visualization of the LC signal can be optimized for future studies.

### 4.1 Limitations of the study

Although this study benefited from a large sample size (n = 605) and therefore greater statistical power compared with previous studies of the LC, there were also some limitations. This was a cross-sectional study reporting on interindividual age-related differences in LC CR, which supports but does not prove that age-related changes occur within individuals, as this would require a longitudinal study. The resolution of the Cam-CAN MT-weighted images (original voxel size 1.5 mm isotropic) was relatively low compared with previous MT-weighted studies (typically 0.39 × 0.39 × 3 mm to 0.7 × 0.7 × 0.7 mm) (Chen et al., 2014, Langley et al., 2016, Priovoulos et al., 2017), which limited the precision of segmentation and may have contributed to some of the variability of LC signal intensity values. Although we statistically corrected the regression models for the differences in TR, it is possible that this may not have entirely accounted for the effect of these differences, which may have influenced the results. However, as the TR differences affected all age groups, these may not have changed the overall pattern of LC CR across the life span. Interindividual differences in head motion may also have contributed to the higher variance seen in older adults, and this would have benefitted from measures of test-retest reliability. Although we obtained LC volume and size estimates that were close to previously published postmortem values (in contrast to previous in vivo MRI studies in healthy adults that have reported LC volumes of less than 30 mm^3^ [Liu et al., 2017]), the voxel number analyses should be interpreted cautiously, as partial volume artifacts characteristic of low resolution acquisitions would have limited the accuracy of LC volume measurements. As the volume of the LC obtained in this study was slightly higher than previously published ex vivo dimensions (85 mm^3^ versus 73 mm^3^ for each LC) (Fernandes et al., 2012), we cannot fully exclude the possibility that the LC mask did not include the pre-LC (or pericoeruleus, which lies rostral to the LC). Our LC mask did not extend as far caudally as did two previously published LC masks (Betts et al., 2017, Keren et al., 2009), so it is likely to have covered the rostral and middle but not the caudal region of the LC. This study found that in a healthy population, LC CR no longer increases with age after around 60 years, but we can only speculate on the biological process or processes underlying this observation (e.g., LC cell loss), as postmortem validation is not available and the precise source of the neuromelanin contrast is unclear. Despite these limitations, our study emphasizes the importance of further investigating LC signal intensity as a potential biomarker of LC-noradrenergic integrity in the over 60s. It would be valuable to subsequently assess the cognitive and behavioral correlates of LC signal intensity in this sample, and obtain follow-up information on any emergent diagnoses of dementia.

## 5 Conclusions

This study found age-related differences in normalized LC signal intensity values, that is, LC CR, on MT-weighted MRI scans in a cross-sectional sample of 605 healthy adults across the life span. These scans were obtained from an open-access database (Cam-CAN) that did not specifically use imaging parameters to investigate the LC and provide insights on how neuromelanin contrast and scanning parameters can be optimized for future studies. A quadratic relationship was observed between LC CR and age, the peak occurring around 60 years, with only the rostral region showing significant age-related decline. There were no differences between males and females. The neurobiological basis and the functional and clinical implications of the observed age-related differences in older adults require further investigation to improve our understanding of the LC-noradrenergic system in healthy aging and neurodegenerative disease.

## Disclosure

The authors have no actual or potential conflicts of interest.

## Appendix A Supplementary data

Supplemental material LC

Liu_LC_CamCAN_MNI

## References

[bib1] Avants B.B., Tustison N.J., Song G., Cook P.A., Klein A., Gee J.C. (2011). A reproducible evaluation of ANTs similarity metric performance in brain image registration. Neuroimage.

[bib2] Betts M.J., Cardenas-Blanco A., Kanowski M., Jessen F., Düzel E. (2017). In vivo MRI assessment of the human locus coeruleus along its rostrocaudal extent in young and older adults. Neuroimage.

[bib3] Braak H., Del Tredici K., Rüb U., de Vos R.A.I., Jansen Steur E.N.H., Braak E. (2003). Staging of brain pathology related to sporadic Parkinson’s disease. Neurobiol. Aging.

[bib4] Braak H., Thal D.R., Ghebremedhin E., Del Tredici K. (2011). Stages of the pathologic process in Alzheimer disease: age categories from 1 to 100 years. J. Neuropathol. Exp. Neurol..

[bib5] Chan-Palay V., Asan E. (1989). Quantitation of catecholamine neurons in the locus coeruleus in human brains of normal young and older adults and in depression. J. Comp. Neurol..

[bib6] Chan-Palay V., Asan E. (1989). Alterations in catecholamine neurons of the locus coeruleus in senile dementia of the Alzheimer type and in Parkinson’s disease with and without dementia and depression. J. Comp. Neurol..

[bib7] Chen X., Huddleston D.E., Langley J., Ahn S., Barnum C.J., Factor S.A., Levey A.I., Hu X. (2014). Simultaneous imaging of locus coeruleus and substantia nigra with a quantitative neuromelanin MRI approach. Magn. Reson. Imaging.

[bib8] Clewett D.V., Lee T.-H., Greening S., Ponzio A., Margalit E., Mather M. (2016). Neuromelanin marks the spot: identifying a locus coeruleus biomarker of cognitive reserve in healthy aging. Neurobiol. Aging.

[bib9] Dahl M.J., Mather M., Duezel S., Bodammer N.C., Lindenberger U., Kuehn S., Werkle-Bergner M. (2018). Locus Coeruleus Integrity Preserves Memory Performance across the Adult Life Span.

[bib10] Dienes Z. (2014). Using Bayes to get the most out of non-significant results. Front. Psychol..

[bib11] Fernandes P., Regala J., Correia F., Gonçalves-Ferreira A.J. (2012). The human locus coeruleus 3-D stereotactic anatomy. Surg. Radiol. Anat..

[bib12] German D.C., Manaye K.F., White C.L., Woodward D.J., McIntire D.D., Smith W.K., Kalaria R.N., Mann D.M. (1992). Disease-specific patterns of locus coeruleus cell loss. Ann. Neurol..

[bib13] German D.C., Walker B.S., Manaye K., Smith W.K., Woodward D.J., North A.J. (1988). The human locus coeruleus: computer reconstruction of cellular distribution. J. Neurosci..

[bib14] Grudzien A., Shaw P., Weintraub S., Bigio E., Mash D.C., Mesulam M.M. (2007). Locus coeruleus neurofibrillary degeneration in aging, mild cognitive impairment and early Alzheimer’s disease. Neurobiol. Aging.

[bib15] Hämmerer D., Callaghan M.F., Hopkins A., Kosciessa J., Betts M., Cardenas-Blanco A., Kanowski M., Weiskopf N., Dayan P., Dolan R.J., Düzel E. (2018). Locus coeruleus integrity in old age is selectively related to memories linked with salient negative events. Proc. Natl. Acad. Sci. U. S. A..

[bib16] Kelly S.C., He B., Perez S.E., Ginsberg S.D., Mufson E.J., Counts S.E. (2017). Locus coeruleus cellular and molecular pathology during the progression of Alzheimer’s disease. Acta Neuropathol. Commun..

[bib17] Keren N.I., Lozar C.T., Harris K.C., Morgan P.S., Eckert M.A. (2009). In vivo mapping of the human locus coeruleus. Neuroimage.

[bib18] Keren N.I., Taheri S., Vazey E.M., Morgan P.S., Granholm A.-C.E., Aston-Jones G.S., Eckert M.A. (2015). Histologic validation of locus coeruleus MRI contrast in post-mortem tissue. Neuroimage.

[bib19] Langley J., Huddleston D.E., Liu C.J., Hu X. (2016). Reproducibility of locus coeruleus and substantia nigra imaging with neuromelanin sensitive MRI. MAGMA.

[bib20] Liu K.Y., Marijatta F., Hämmerer D., Acosta-Cabronero J., Düzel E., Howard R.J. (2017). Magnetic resonance imaging of the human locus coeruleus: a systematic review. Neurosci. Biobehav. Rev..

[bib21] Loughlin S.E., Foote S.L., Bloom F.E. (1986). Efferent projections of nucleus locus coeruleus: topographic organization of cells of origin demonstrated by three-dimensional reconstruction. Neuroscience.

[bib22] Manaye K.F., McIntire D.D., Mann D.M., German D.C. (1995). Locus coeruleus cell loss in the aging human brain: a non-random process. J. Comp. Neurol..

[bib23] Mann D.M.A., Yates P.O. (1974). Lipoprotein pigments—their relationship to ageing in the human nervous system II. The melanin content of pigmented nerve cells. Brain.

[bib24] Morey R.D., Rouder J.N. (2011). Bayes factor approaches for testing interval null hypotheses. Psychol. Methods.

[bib25] Mouton P.R., Pakkenberg B., Gundersen H.J., Price D.L. (1994). Absolute number and size of pigmented locus coeruleus neurons in young and aged individuals. J. Chem. Neuroanat..

[bib26] Nakane T., Nihashi T., Kawai H., Naganawa S. (2008). Visualization of neuromelanin in the Substantia nigra and locus ceruleus at 1.5T using a 3D-gradient echo sequence with magnetization transfer contrast. Magn. Reson. Med. Sci..

[bib27] Ohm T.G., Busch C., Bohl J. (1997). Unbiased estimation of neuronal numbers in the human nucleus coeruleus during aging. Neurobiol. Aging.

[bib28] Priovoulos N., Jacobs H.I.L., Ivanov D., Uludag K., Verhey F.R.J., Poser B.A. (2017). High-resolution in vivo imaging of human locus coeruleus by magnetization transfer MRI at 3T and 7T. Neuroimage.

[bib29] Samuels E.R., Szabadi E. (2008). Functional neuroanatomy of the noradrenergic locus coeruleus: its roles in the regulation of arousal and autonomic function part I: principles of functional organisation. Curr. Neuropharmacol..

[bib30] Samuels E.R., Szabadi E. (2008). Functional neuroanatomy of the noradrenergic locus coeruleus: its roles in the regulation of arousal and autonomic function part II: physiological and pharmacological manipulations and pathological alterations of locus coeruleus activity in humans. Curr. Neuropharmacol..

[bib31] Sara S.J. (2009). The locus coeruleus and noradrenergic modulation of cognition. Nat. Rev. Neurosci..

[bib32] Sasaki M., Shibata E., Kudo K., Tohyama K. (2008). Neuromelanin-sensitive MRI. Clin. Neuroradiol..

[bib33] Sasaki M., Shibata E., Tohyama K., Takahashi J., Otsuka K., Tsuchiya K., Takahashi S., Ehara S., Terayama Y., Sakai A. (2006). Neuromelanin magnetic resonance imaging of locus ceruleus and substantia nigra in Parkinson’s disease. Neuroreport.

[bib34] Schwarz L.A., Luo L. (2015). Organization of the locus coeruleus-norepinephrine system. Curr. Biol..

[bib35] Shafto M.A., Tyler L.K., Dixon M., Taylor J.R., Rowe J.B., Cusack R., Calder A.J., Marslen-Wilson W.D., Duncan J., Dalgleish T., Henson R.N., Brayne C., Matthews F.E., Cam-CAN (2014). The Cambridge Centre for Ageing and Neuroscience (Cam-CAN) study protocol: a cross-sectional, lifespan, multidisciplinary examination of healthy cognitive ageing. BMC Neurol..

[bib36] Shibata E., Sasaki M., Tohyama K., Kanbara Y., Otsuka K., Ehara S., Sakai A. (2006). Age-related changes in locus ceruleus on neuromelanin magnetic resonance imaging at 3 Tesla. Magn. Reson. Med. Sci..

[bib37] Simonsohn U. (2018). Two-lines: A Valid Alternative to the Invalid Testing of U-shaped Relationships with Quadratic Regressions. https://ssrn.com/abstract=3021690.

[bib38] Taylor J.R., Williams N., Cusack R., Auer T., Shafto M.A., Dixon M., Tyler L.K., Cam-Can, Henson R.N. (2017). The Cambridge Centre for Ageing and Neuroscience (Cam-CAN) data repository: structural and functional MRI, MEG, and cognitive data from a cross-sectional adult lifespan sample. Neuroimage.

[bib39] Theofilas P., Ehrenberg A.J., Dunlop S., Di Lorenzo Alho A.T., Nguy A., Leite R.E.P., Rodriguez R.D., Mejia M.B., Suemoto C.K., Ferretti-Rebustini R.E.D.L., Polichiso L., Nascimento C.F., Seeley W.W., Nitrini R., Pasqualucci C.A., Jacob Filho W., Rueb U., Neuhaus J., Heinsen H., Grinberg L.T. (2017). Locus coeruleus volume and cell population changes during Alzheimer’s disease progression: a stereological study in human postmortem brains with potential implication for early-stage biomarker discovery. Alzheimers. Dement..

[bib40] Tona K.-D., Keuken M.C., de Rover M., Lakke E., Forstmann B.U., Nieuwenhuis S., van Osch M.J.P. (2017). In vivo visualization of the locus coeruleus in humans: quantifying the test-retest reliability. Brain Struct. Funct..

[bib41] Trujillo P., Summers P.E., Ferrari E., Zucca F.A., Sturini M., Mainardi L.T., Cerutti S., Smith A.K., Smith S.A., Zecca L., Costa A. (2016). Contrast mechanisms associated with neuromelanin-MRI. Magn. Reson. Med..

[bib42] Tustison N.J., Avants B.B., Cook P.A., Zheng Y., Egan A., Yushkevich P.A., Gee J.C. (2010). N4ITK: improved N3 bias correction. IEEE Trans. Med. Imaging.

[bib43] Ungerstedt U. (1971). Stereotaxic mapping of the monoamine pathways in the rat brain. Acta Physiol. Scand. Suppl..

[bib44] Yushkevich P.A., Piven J., Hazlett H.C., Smith R.G., Ho S., Gee J.C., Gerig G. (2006). User-guided 3D active contour segmentation of anatomical structures: significantly improved efficiency and reliability. Neuroimage.

[bib45] Zarow C., Lyness S.A., Mortimer J.A., Chui H.C. (2003). Neuronal loss is greater in the locus coeruleus than nucleus basalis and substantia nigra in Alzheimer and Parkinson diseases. Arch. Neurol..

[bib46] Zecca L., Casella L., Albertini A., Bellei C., Zucca F.A., Engelen M., Zadlo A., Szewczyk G., Zareba M., Sarna T. (2008). Neuromelanin can protect against iron-mediated oxidative damage in system modeling iron overload of brain aging and Parkinson’s disease. J. Neurochem..

[bib47] Zucca F.A., Bellei C., Giannelli S., Terreni M.R., Gallorini M., Rizzio E., Pezzoli G., Albertini A., Zecca L. (2006). Neuromelanin and iron in human locus coeruleus and substantia nigra during aging: consequences for neuronal vulnerability. J. Neural Transm..

