# Corrigendum to in vivo visualization of age-related differences in the locus coeruleus Neurobiology of Aging Volume 74, February 2019, Pages 101–111

**DOI:** 10.1016/j.neurobiolaging.2020.03.004

**Published:** 2020-07

**Authors:** Kathy Y. Liu, Julio Acosta-Cabronero, Arturo Cardenas-Blanco, Clare Loane, Alex J. Berry, Matthew J. Betts, Rogier A. Kievit, Richard N. Henson, Emrah Düzel, Cam-CAN, Robert Howard, Dorothea Hämmerer

**Affiliations:** a Division of Psychiatry, University College London, London, UK; b Wellcome Centre for Human Neuroimaging, UCL Institute of Neurology, University College London, London, UK; c German Center for Neurodegenerative Diseases (DZNE), Magdeburg, Germany; d Institute of Cognitive Neurology and Dementia Research, Otto-von-Guericke-University Magdeburg, Magdeburg, Germany; e Institute of Cognitive Neuroscience, University College London, London, UK; f Camden & Islington NHS Foundation Trust, London, UK; g Medical Research Council Cognition and Brain Sciences Unit, University of Cambridge, Cambridge, UK; h Cambridge Centre for Ageing and Neuroscience (Cam-CAN), University of Cambridge and MRC Cognition and Brain Sciences Unit, Cambridge, UK

The authors regret to inform readers that the following errors were detected in the original article.

Two figure legends (Fig. 5, panel B legend and Fig. 6, panel D legend) have been corrected and now match the main text.

Fig. 5, panel B legend.

**Figure:**
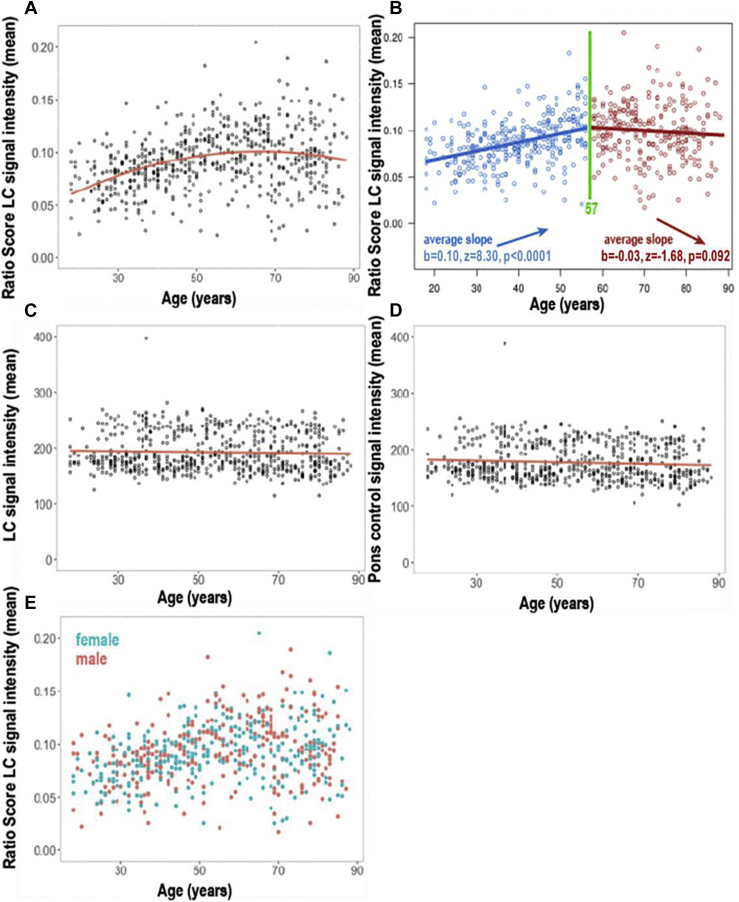

Fig. 6, panel D legend.

**Figure:**
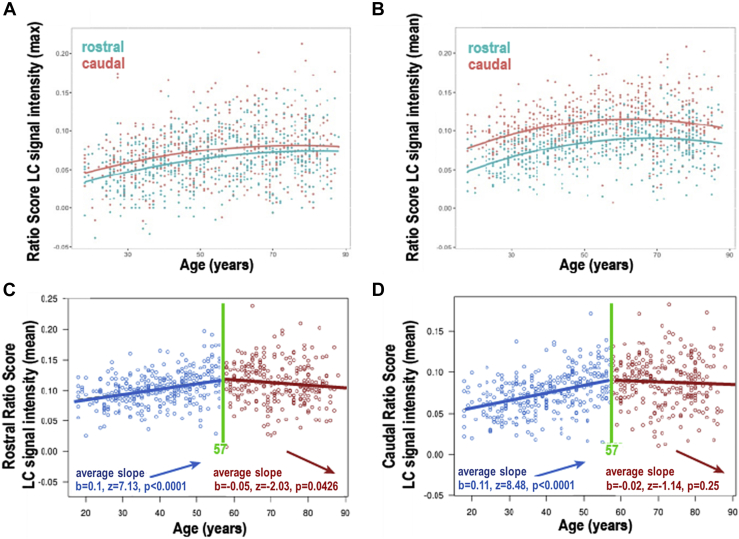

The LC Cam-CAN mask spatial overlap relative to the Betts mask should be 51%, not 19% as reported, due to a data analysis coding error.

The correct Cam-CAN LC mask can be found here: https://osf.io/r2bwk/. The supplementary zip file titled ‘Liu_LC_CamCAN_MNI’ should not be used as it is a version of the Dahl et al., 2019 mask (https://www.mpib-berlin.mpg.de/lc-map), which was mislabeled and uploaded in error during the submission process. Specifically, it is a binarized version of the probabilistic BASE-II LC mask with slightly fewer voxels (164 vs 175 voxels).

The reported LC signal intensity values and the main conclusions of the study are unaffected. The authors apologize for any inconvenience caused.

