# Supplementary material for: In vivo visualization of age-related differences in the locus coeruleus
Source: Neurobiol Aging. 2019 Feb;74:101–11. doi: 10.1016/j.neurobiolaging.2018.10.014 (PMC6338679; doi:10.1016/j.neurobiolaging.2018.10.014)
Supplement: Supplemental material LC [file mmc1.docx]

To explore whether a different reference region would change our findings, we segmented a larger 3D pontine reference region (the new pons mask consisted of 144 voxels) consisting of right and left subregions (see Supplemental Figure 1). There was no significant relationship between age and signal intensity in this reference region (r=-0.03, p=0.45), and the right pontine reference region had a higher raw mean signal intensity than the left (t(604)=-17.4, p<0.001), in contrast to the LC which showed higher raw mean and CR values in left vs right. When signal intensity values in this larger reference region were used to obtain normalized LC signal intensity (LC CR) values, similar results to our initial data were obtained after excluding extreme outlier values (which can be expected to be more frequent in a larger mask that encompasses more heterogeneous tissues; 3 negative values and 11 values above 0.3). As previously, age-related differences in LC CR were better explained by a quadratic model (adjusted R^2^ = 0.02, p=0.002) (Supplemental Figure 2) than a linear model (adj R^2^ =0.0004 , p = 0.26). A subsequent two-lines test (Supplemental Figure 2) showed that both the upwards and downwards slopes were significant (p=0.02 and p=0.006 respectively).


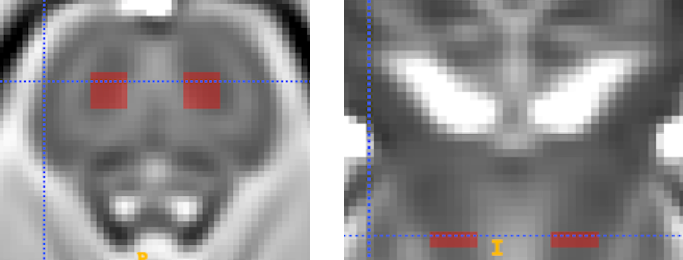


Supplemental Figure 1: The alternative pontine reference region mask (red) showing axial (left) and coronal (right) views.


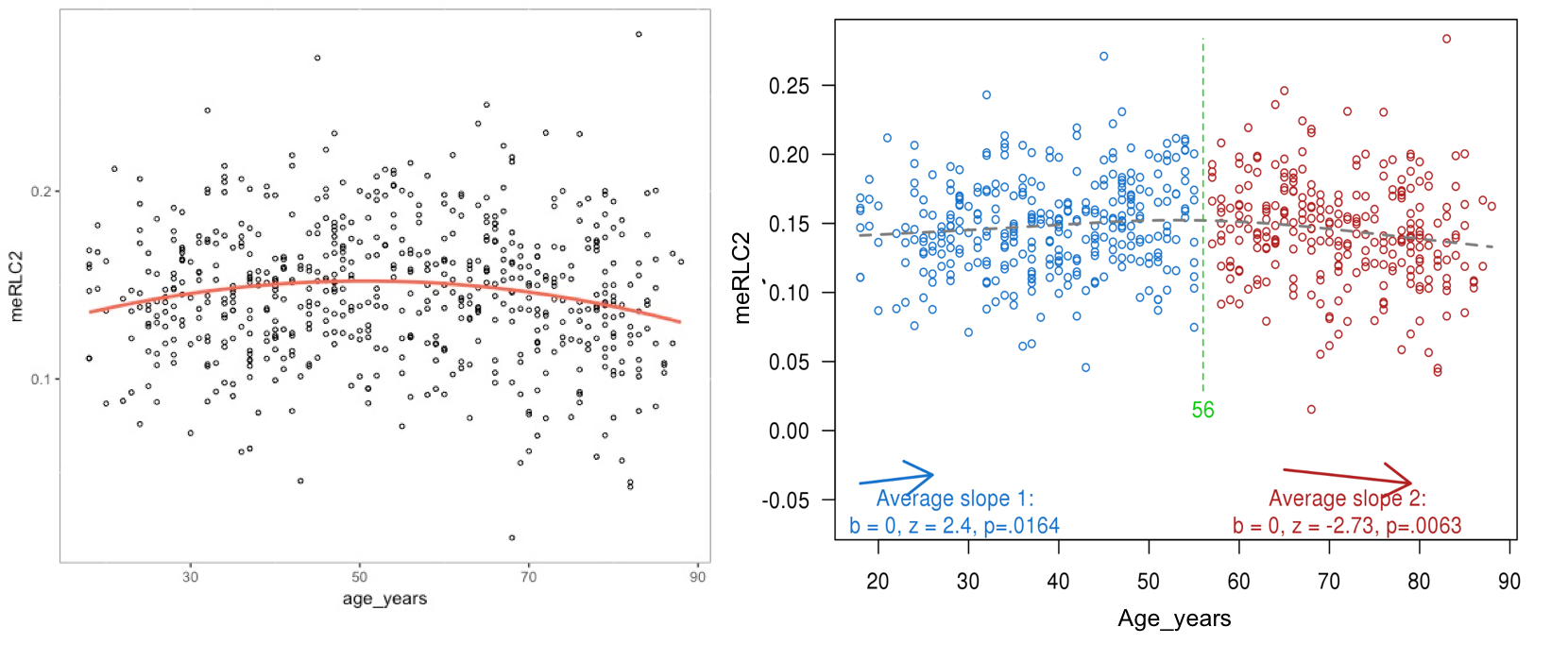


Supplemental Figure 2: Plots showing mean LC CR vs age using the alternative reference region, and a subsequent the two-lines test (right).
